# Supplementary material for: Analysing similarities between legal court documents using natural language processing approaches based on transformers
Source: PLoS One. 2025 Apr 8;20(4):e0320244. doi: 10.1371/journal.pone.0320244 (PMC11978053; doi:10.1371/journal.pone.0320244)
Supplement: S1 Table — Sabiá Jud. group name. Most relevant TPU terms for all groups generated from Sabiá Jud. model. (PDF) [file pone.0320244.s001.pdf]

**S1 Table.** Most relevant TPU terms for all groups generated from Sabiá Jud. model.

| Group id | Amount of documents | Average similarity | Group name (TPUs) | Cosine similarity |
|---|---|---|---|---|
| 0 | 8,435 | 0.999998 | sucessão de empregadores | 0.999999 |
| | | | execução provisória | 0.999998 |
| | | | indenizado efeitos | 0.999998 |
| 1 | 1,238 | 0.999485 | responsabilidade solidária subsidiária | 0.999425 |
| | | | sucumbência | 0.999423 |
| | | | direito de greve | 0.999422 |
| 2 | 5,977 | 0.999998 | nomeação à autoria | 0.999999 |
| | | | entes públicos | 0.999999 |
| | | | prova ilícita | 0.999998 |
| 3 | 6,902 | 0.999998 | direito de greve | 0.999999 |
| | | | responsabilidade solidária subsidiária | 0.999998 |
| | | | sucumbência | 0.999998 |
| 4 | 175 | 0.999929 | ctps | 0.99992 |
| | | | produção | 0.999916 |
| | | | astreintes | 0.999911 |
| 5 | 2,916 | 0.999994 | agravo de instrumento | 0.999999 |
| | | | deficiente físico | 0.999999 |
| | | | cesta básica | 0.999998 |
| 6 | 449 | 0.998100 | conexão | 0.991685 |
| | | | recurso de revista | 0.991684 |
| | | | extinção do processo sem resolução de mérito | 0.991684 |
| 7 | 49,638 | 1.000000 | ctps | 0.999804 |
| | | | período de graça | 0.999796 |
| | | | astreintes | 0.999795 |
| 8 | 2,689 | 0.999999 | licença previdenciária | 0.999999 |
| | | | ação de cobrança | 0.999999 |
| | | | descontos previdenciários | 0.999998 |
| 9 | 829 | 0.997124 | tempo de exposição | 0.987773 |
| | | | fase de execução | 0.987773 |
| | | | jus postulandi | 0.98777 |
| 10 | 3,861 | 0.999999 | licença previdenciária | 0.999999 |
| | | | suspeição | 0.999998 |
| | | | descontos previdenciários | 0.999998 |
| 11 | 6,099 | 0.999996 | trabalhador eventual | 0.999999 |
| | | | representante comercial autônomo | 0.999999 |
| | | | multa por descumprimento de ordem judicial | 0.999998 |
| 12 | 1,148 | 0.988145 | remessa necessária | 0.970753 |
| | | | jornalistas | 0.970744 |
| | | | processo do trabalho | 0.970744 |
| 13 | 6,284 | 0.999999 | agravo de instrumento | 0.999999 |
| | | | cesta básica | 0.999999 |
| | | | compensação em atividade insalubre | 0.999999 |
| 14 | 3,822 | 0.999992 | acúmulo de função | 0.999999 |
| | | | perícia local de trabalho | 0.999999 |
| | | | auxílio creche | 0.999999 |

| Group id | Amount of documents | Average similarity | Group name (TPUs) | Cosine similarity |
|---|---|---|---|---|
| 15 | 392 | 0.999900 | concessão de serviço público | 0.999998 |
| | | | administração pública | 0.999998 |
| | | | portuário | 0.999998 |
| 16 | 3,785 | 0.999999 | ente público | 0.999999 |
| | | | dirigente sindical | 0.999999 |
| | | | cálculo repercussão | 0.999999 |
| 17 | 1,718 | 0.999969 | ctps | 0.999800 |
| | | | astreintes | 0.999790 |
| | | | período de graça | 0.999789 |
| 18 | 6,564 | 0.999997 | responsabilidade solidária subsidiária | 0.999999 |
| | | | sucumbência | 0.999999 |
| | | | indenizado efeitos | 0.999998 |
| 19 | 1,232 | 0.999376 | ctps | 0.999202 |
| | | | astreintes | 0.999197 |
| | | | imposto de renda | 0.999194 |
| 20 | 322 | 0.941031 | recuperação judicial | 0.477418 |
| | | | jornalistas | 0.477411 |
| | | | auxílio creche | 0.477406 |
| 21 | 5,793 | 0.999999 | seguro de vida | 0.999998 |
| | | | atraso na audiência | 0.999997 |
| | | | sucessão de empregadores | 0.999997 |
| 22 | 1,110 | 0.999348 | acúmulo de função | 0.999068 |
| | | | contrato por prazo determinado | 0.999067 |
| | | | capacidade processual | 0.999067 |
| 23 | 5,947 | 0.999999 | suspeição | 0.999999 |
| | | | licença previdenciária | 0.999998 |
| | | | deficiente físico | 0.999998 |
| 24 | 4,962 | 0.999999 | direito de greve | 0.999999 |
| | | | remuneração, verbas indenizatórias e benefícios | 0.999999 |
| | | | ação de cobrança | 0.999999 |
| 25 | 1,605 | 0.999753 | ctps | 0.999687 |
| | | | período de graça | 0.999682 |
| | | | astreintes | 0.99968 |
| 26 | 2,709 | 0.999999 | promoção | 0.999999 |
| | | | bancos | 0.999999 |
| | | | perícia | 0.999999 |
| 27 | 554 | 0.993318 | reajuste salarial | 0.964362 |
| | | | imposto de renda | 0.964357 |
| | | | litigância de má-fé | 0.964344 |
| 28 | 4,825 | 0.999999 | ação cautelar | 0.999999 |
| | | | compensação em atividade insalubre | 0.999999 |
| | | | tomador de serviços terceirização | 0.999998 |
| 29 | 790 | 0.99999 | ctps | 0.999862 |
| | | | tarefa | 0.999851 |
| | | | astreintes | 0.999851 |

| Group id | Amount of documents | Average similarity | Group name (TPUs) | Cosine similarity |
|---|---|---|---|---|
| 30 | 746 | 0.998293 | parcela incontroversa | 0.996976 |
| | | | cláusula penal | 0.996974 |
| | | | gratificação de função | 0.996974 |
| 31 | 5,731 | 0.999997 | trabalhador avulso | 0.999999 |
| | | | adicional de transferência | 0.999999 |
| | | | reconhecimento de relação de emprego | 0.999998 |
| 32 | 6,286 | 0.999998 | violação literal à disposição de lei | 0.999999 |
| | | | repetição da prova | 0.999998 |
| | | | contrato de trabalho temporário | 0.999998 |
| 33 | 925 | 0.998547 | reajuste salarial | 0.997004 |
| | | | decadência | 0.997001 |
| | | | adicional de risco | 0.996999 |
| 34 | 2,788 | 0.999999 | salário família | 0.999999 |
| | | | salário in natura | 0.999999 |
| | | | extinção normal do contrato a termo | 0.999998 |
| 35 | 1,347 | 0.99995 | parcela incontroversa | 0.999998 |
| | | | adicional de confinamento | 0.999997 |
| | | | gratificação de função | 0.999997 |
| 36 | 1,587 | 0.996753 | fase de execução | 0.989751 |
| | | | tempo de exposição | 0.989746 |
| | | | jus postulandi | 0.989746 |
| 37 | 4,322 | 0.999998 | sucumbência | 0.999999 |
| | | | condenação solidária subsidiária | 0.999998 |
| | | | responsabilidade solidária subsidiária | 0.999998 |
| 38 | 4,201 | 0.999999 | deficiente físico | 0.999999 |
| | | | engenheiro, arquiteto e engenheiro agrônomo | 0.999999 |
| | | | agravo de instrumento | 0.999999 |
| 39 | 181 | 0.999065 | fase de execução | 0.999425 |
| | | | tempo de exposição | 0.999412 |
| | | | rescisão do contrato de trabalho | 0.999411 |
| 40 | 4,330 | 0.999995 | reconhecimento de relação de emprego | 0.999999 |
| | | | adicional de transferência | 0.999999 |
| | | | equipamento de proteção individual epi | 0.999999 |
| 41 | 4,056 | 0.999998 | nomeação à autoria | 0.999999 |
| | | | violação literal à disposição de lei | 0.999999 |
| | | | trabalho em condições análogas à de escravo | 0.999999 |
| 42 | 368 | 0.996492 | processo do trabalho | 0.970955 |
| | | | complementação de benefício previdenciário | 0.970953 |
| | | | acidente de trabalho | 0.970951 |
| 43 | 224 | 0.899517 | assistência judiciária gratuita | 0.511948 |
| | | | sucumbência | 0.511935 |
| | | | sucessão | 0.511931 |
| 44 | 1,077 | 0.998866 | responsabilidade solidária subsidiária | 0.997297 |
| | | | direito de greve | 0.997295 |
| | | | sucumbência | 0.997294 |

| Group id | Amount of documents | Average similarity | Group name (TPUs) | Cosine similarity |
|---|---|---|---|---|
| 45 | 3,330 | 0.999998 | ação rescisória | 0.999999 |
| | | | conversão de regime jurídico | 0.999999 |
| | | | aeroviários | 0.999999 |
| 46 | 159 | 0.961141 | acúmulo de função | 0.765710 |
| | | | contrato por prazo determinado | 0.765697 |
| | | | capacidade processual | 0.765693 |
| 47 | 5,250 | 0.999999 | descontos previdenciários | 0.999999 |
| | | | licença previdenciária | 0.999999 |
| | | | ação de cobrança | 0.999998 |
| 48 | 7,341 | 0.999998 | trabalhador avulso | 0.999999 |
| | | | cabimento | 0.999998 |
| | | | adicional de transferência | 0.999998 |
| 49 | 625 | 0.999984 | ctps | 0.999854 |
| | | | produção | 0.999847 |
| | | | tarefa | 0.999843 |
| 50 | 1,395 | 0.99999 | ação cautelar | 0.999998 |
| | | | sucessão | 0.999997 |
| | | | cesta básica | 0.999996 |
| 51 | 3,684 | 0.999998 | ação de cobrança | 0.999999 |
| | | | descontos previdenciários | 0.999999 |
| | | | direito de greve | 0.999999 |
| 52 | 4,108 | 0.999998 | agravo de instrumento | 1.000000 |
| | | | deficiente físico | 0.999999 |
| | | | cesta básica | 0.999999 |
| 53 | 1,885 | 0.999746 | descontos previdenciários | 0.999839 |
| | | | licença previdenciária | 0.999838 |
| | | | forma de cálculo | 0.999838 |
| 54 | 515 | 0.998974 | capacidade processual | 0.998213 |
| | | | acúmulo de função | 0.998213 |
| | | | relação de trabalho | 0.998211 |
