# Supplementary figures and images for: Analysing similarities between legal court documents using natural language processing approaches based on transformers
Source: PLoS One. 2025 Apr 8;20(4):e0320244. doi: 10.1371/journal.pone.0320244 (PMC11978053; doi:10.1371/journal.pone.0320244)

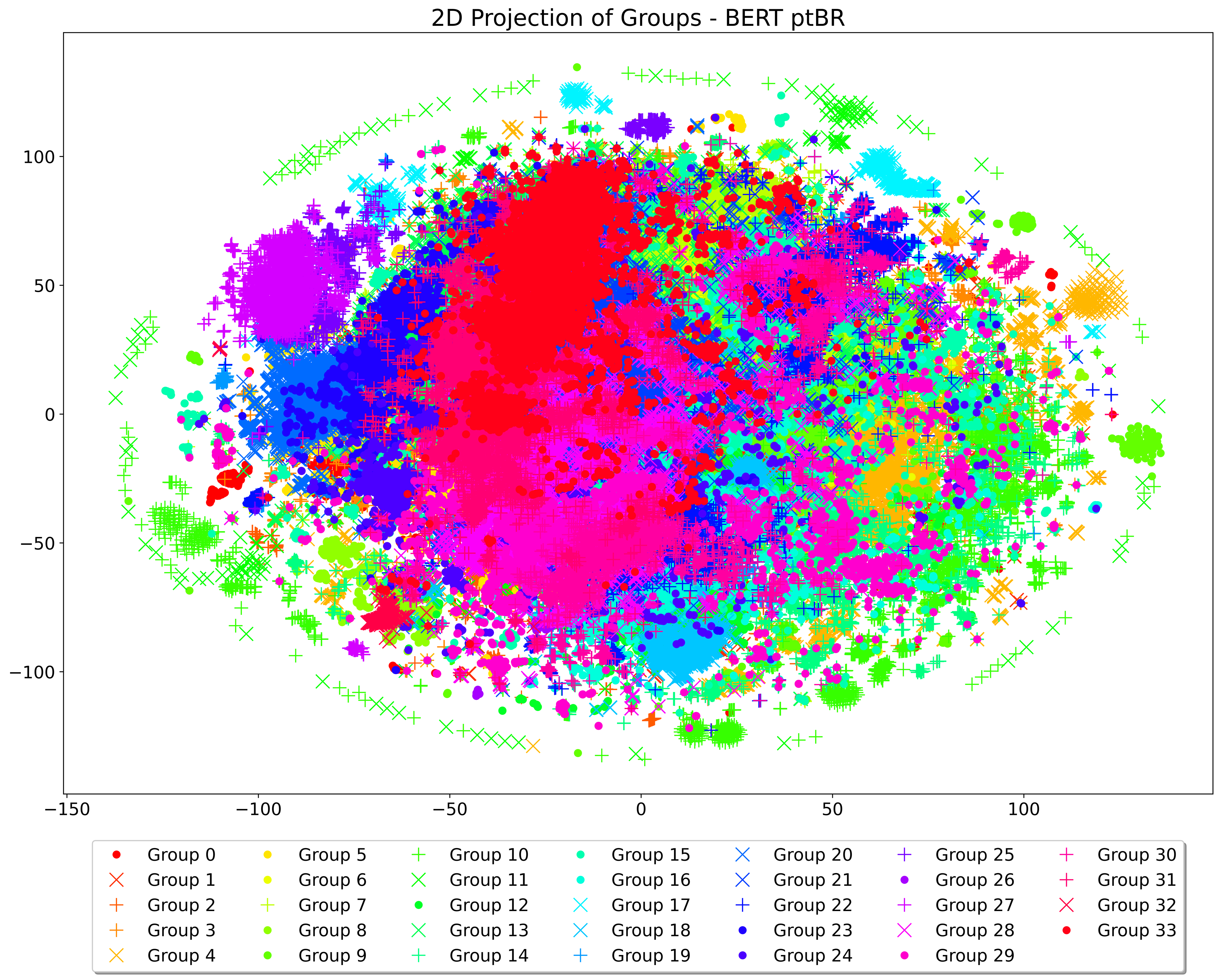

Supplement: S1 Fig — 2D Projection of groups - BERT ptBR. Groups of documents formed using the BERT ptBR. technique, projected in two dimensions based on the test dataset. (TIF) [file pone.0320244.s002.tif]

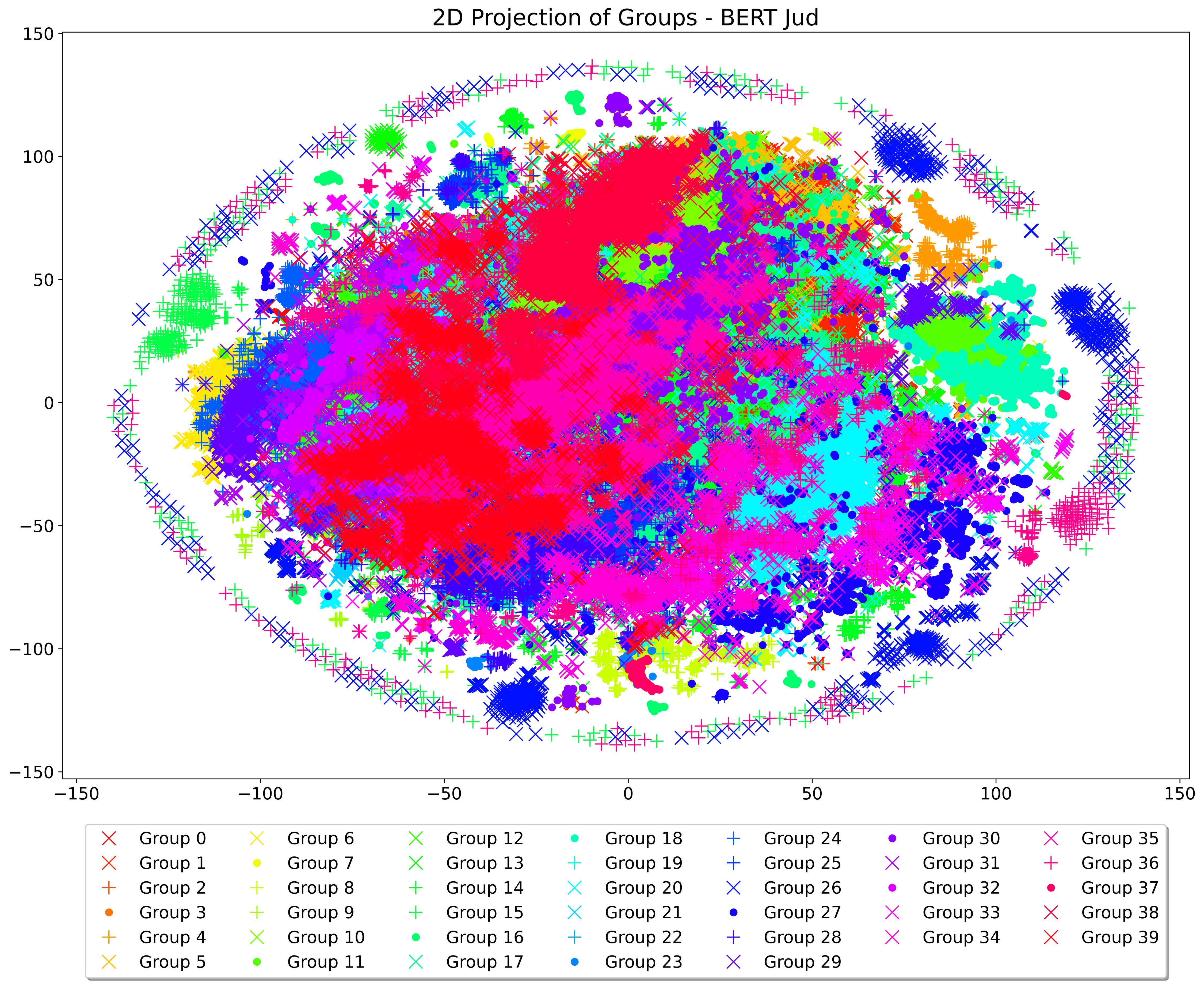

Supplement: S2 Fig — 2D Projection of groups - BERT Jud. Groups of documents formed using the BERT Jud. technique, projected in two dimensions based on the test dataset. (TIF) [file pone.0320244.s003.tif]

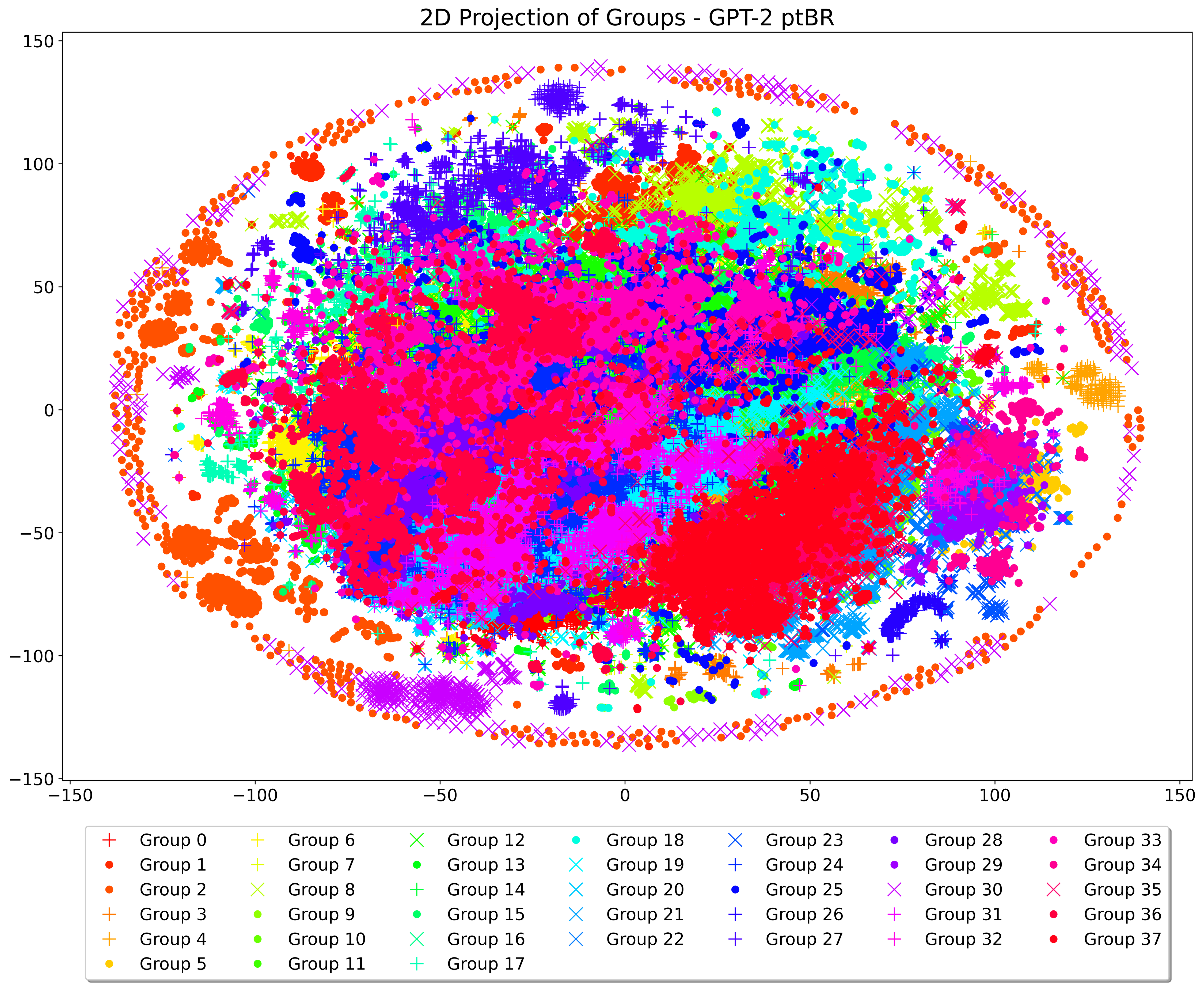

Supplement: S3 Fig — 2D Projection of groups - GPT-2 ptBR. Groups of documents formed using the GPT-2 ptBR. technique, projected in two dimensions based on the test dataset. (TIF) [file pone.0320244.s004.tif]

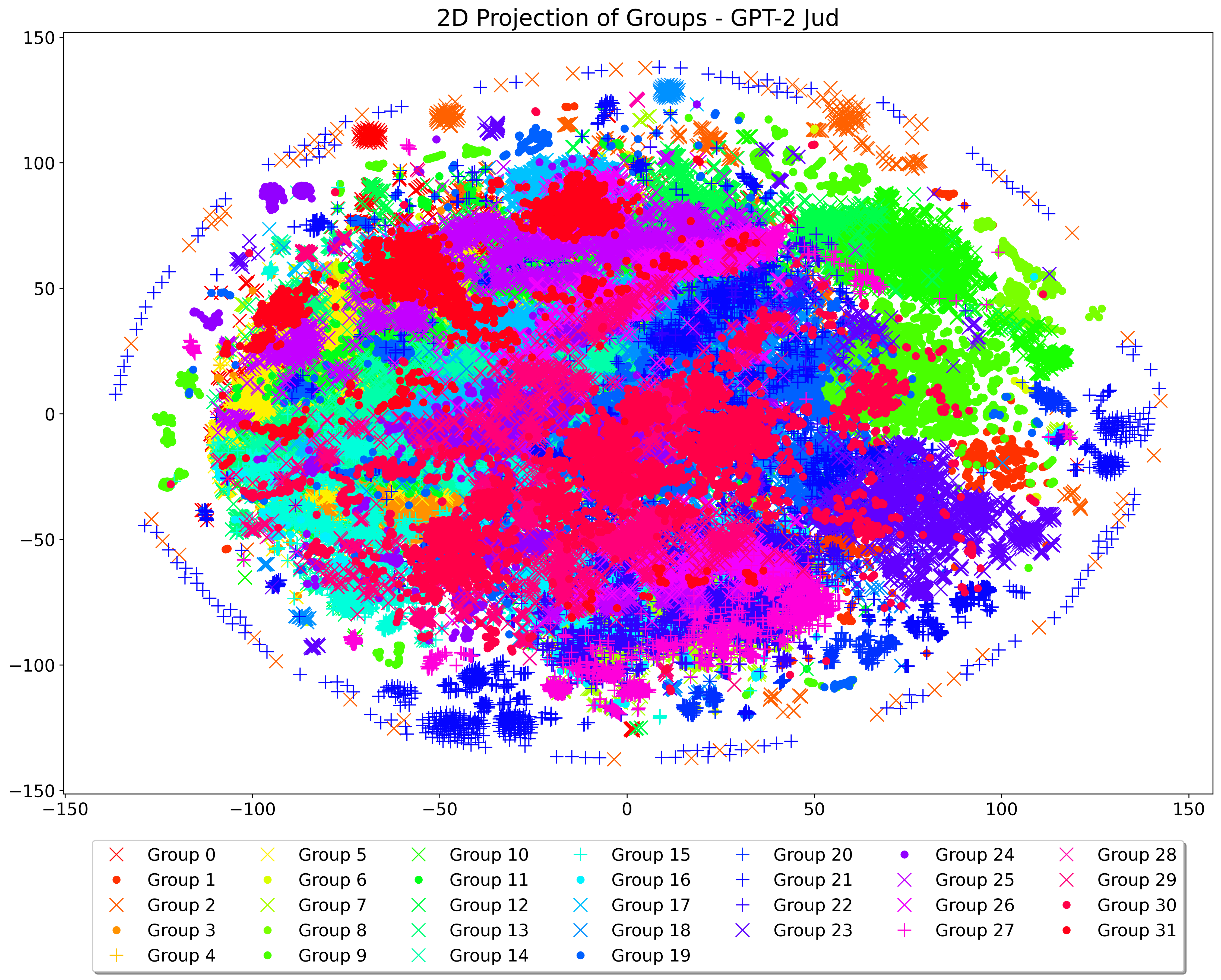

Supplement: S4 Fig — 2D Projection of groups - GPT-2 Jud. Groups of documents formed using the GPT-2 Jud. technique, projected in two dimensions based on the test dataset. (TIF) [file pone.0320244.s005.tif]

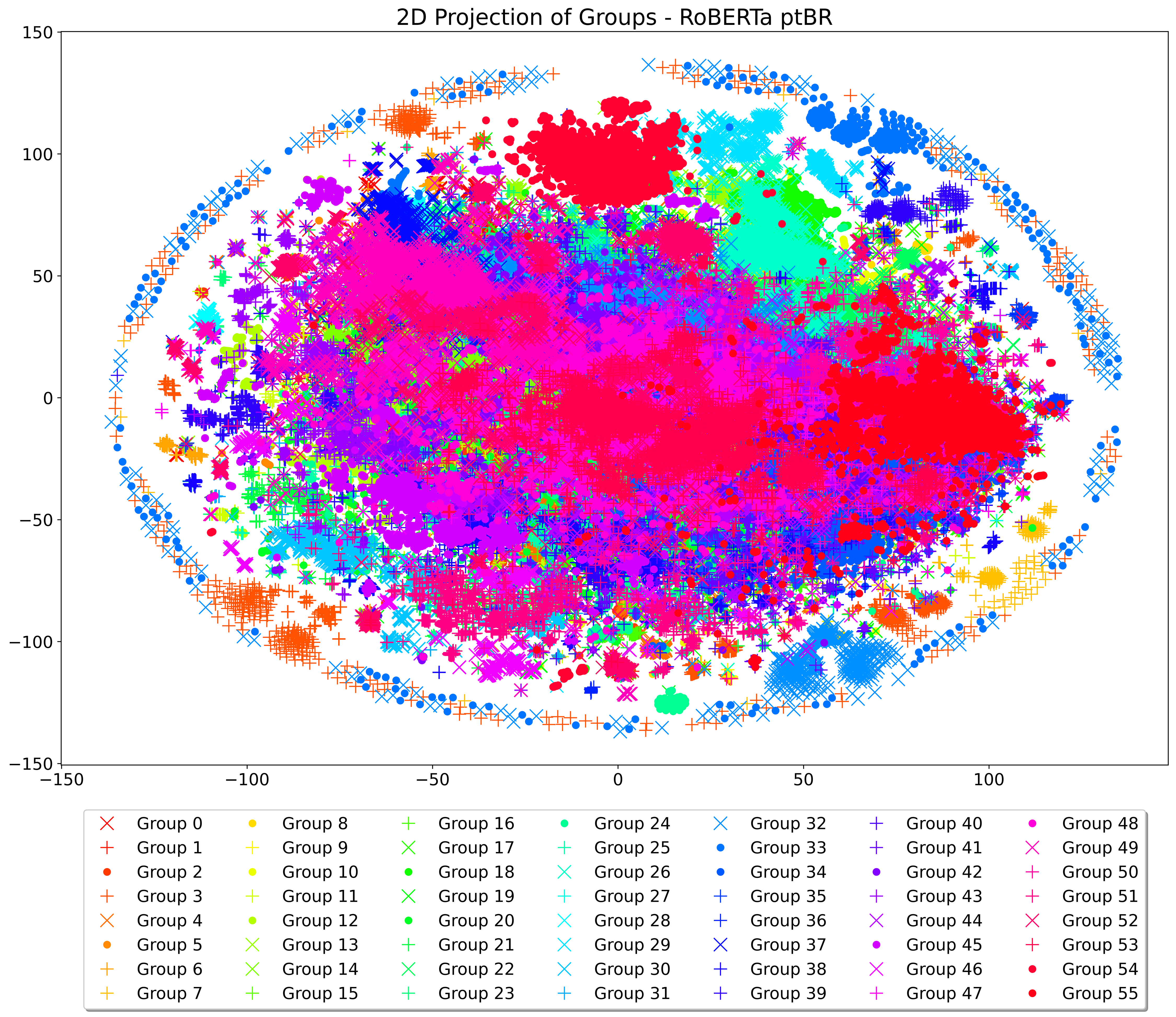

Supplement: S5 Fig — 2D Projection of groups - RoBERTa ptBR. Groups of documents formed using the RoBERTa ptBR. technique, projected in two dimensions based on the test dataset. (TIF) [file pone.0320244.s006.tif]

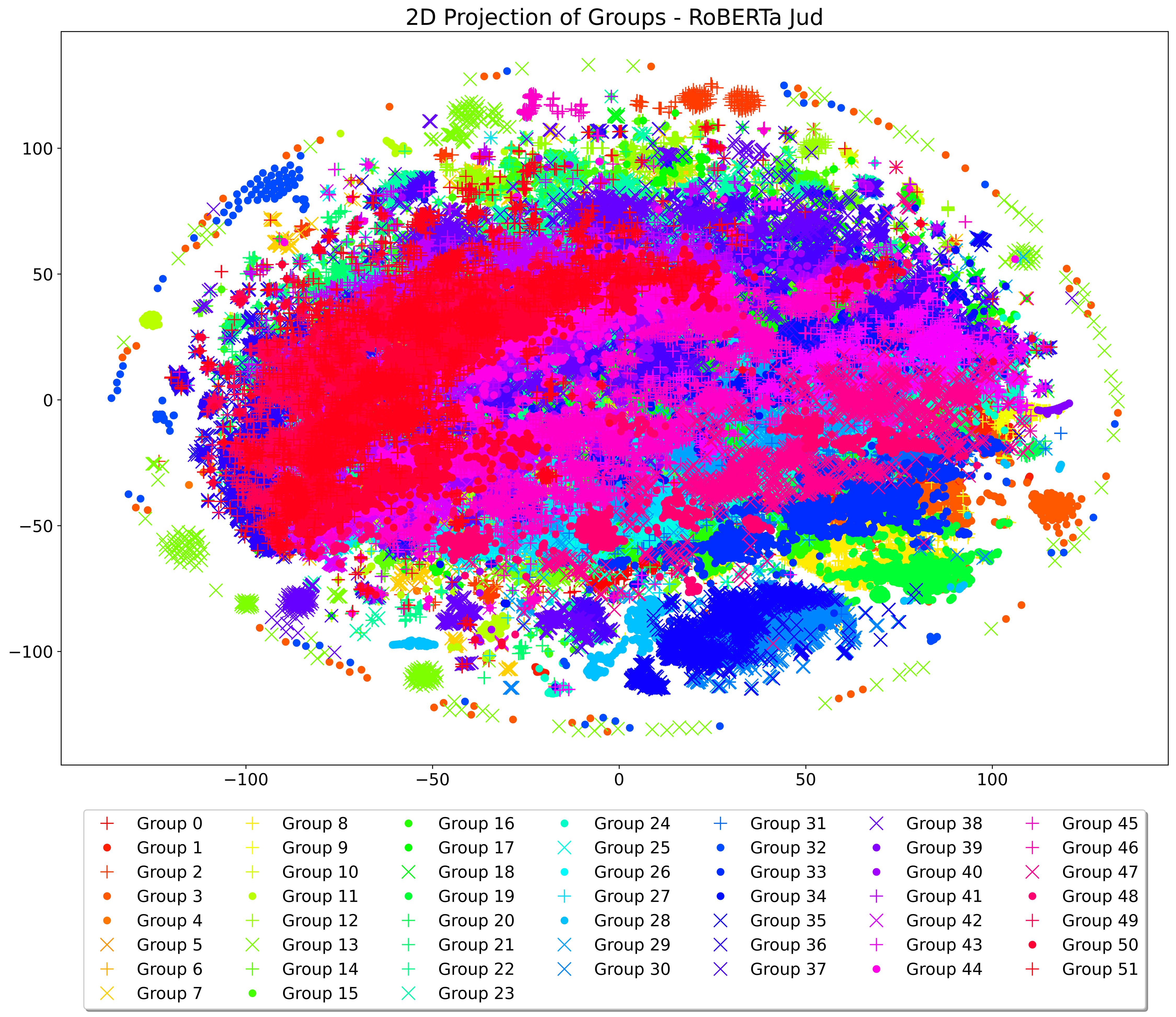

Supplement: S6 Fig — 2D Projection of groups - RoBERTa Jud. Groups of documents formed using the RoBERTa Jud. technique, projected in two dimensions based on the test dataset. (TIF) [file pone.0320244.s007.tif]

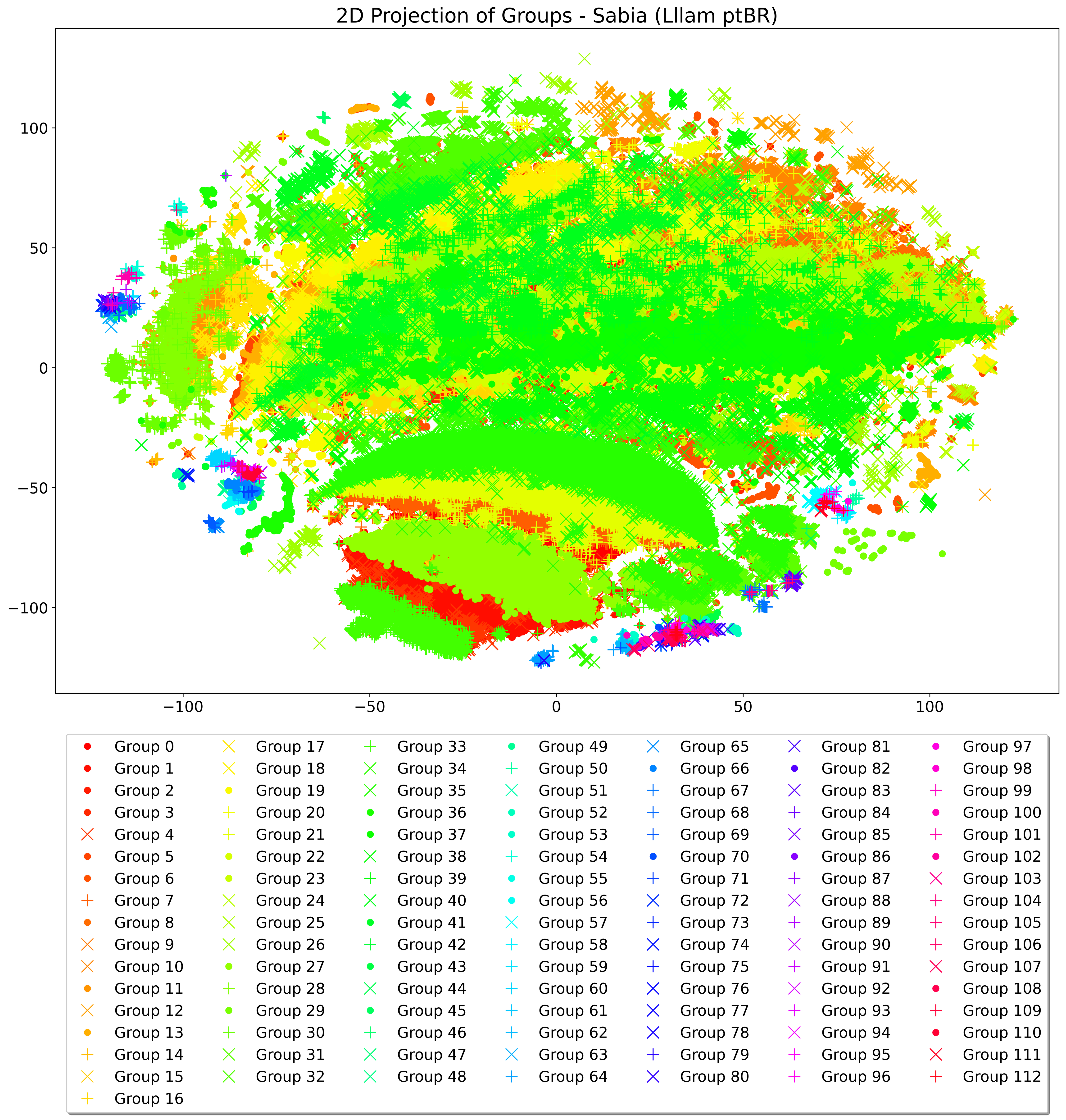

Supplement: S7 Fig — 2D Projection of groups - Sabiá 7B ptBR. Groups of documents formed using the Sabiá 7B ptBR. technique, projected in two dimensions based on the test dataset. (TIF) [file pone.0320244.s008.tif]
