# Supplementary material for: Analysing similarities between legal court documents using natural language processing approaches based on transformers
Source: PLoS One. 2025 Apr 8;20(4):e0320244. doi: 10.1371/journal.pone.0320244 (PMC11978053; doi:10.1371/journal.pone.0320244)
Supplement: Attachment — Submitted filename: Response to Reviewers.pdf [file pone.0320244.s010.pdf]

Firstly, we would like to thank the comments and criticisms about our paper. Right now, we are ready to answer step by step all the major issues and minor corrections according to your requests. We hope these changes and explanations will satisfy all the raised points.

Editor:

This paper clustered 210,000 legal documents from the Brazilian justice system. The authors used k-means clustering on the document embeddings using transformer-based models: BERT, RoBERTa, GPT-2, and Llama. They found that the average within-cluster document similarity increases when they used models fine-tuned to the legal domain.

The paper lacks novelty and its experiments are not strong.

Answer: Thank you for your valuable comment.

Regarding the question concerning the lacks novelty, the following explanation has been added to the "Introduction" section for further clarity:

Page 4 lines 120-131

> *"*
>
> ***Therefore, the main innovations of this work are:***
>
> - ***This research presents a novel analysis of the applicability of transformer-based models for clustering similar documents in the legal field.***
> - ***Unsupervised learning was applied to identify patterns and determine the most effective and representative groups by clustering the embeddings generated by the transformer-based models for each legal document.***
> - ***A comprehensive methodology was developed to ensure the reproducibility of results, enabling its application to not only the legal sector but also other specialised domain applications, for both the tested models and future transformer-based models.***
> - ***The dataset used in this research has been made publicly available to support reproducibility and applicability to future studies in this field and beyond.***
>
> *"*

Regarding the question concerning whether the experiments are not strong, a more detailed examination of the inertia plots was conducted, with a particular focus on the results obtained by the model using Sabiá Jud. The following text was added after the Inertia plots figure in the "Results and Discussion" section to provide further clarity:

Page 12 lines 432-441

> *"*
>
> ***The analysis of the inertia plots presented in Figure 8 reveals that, for all models except Sabiá Jud., the inertia generally decreased as the number of clusters increased, although with significant variance. An asymptotic downward trend can be observed through the dashed lines in these plots. Furthermore, the results emphasise***

that the inertia values obtained for all specialised models (BERT Jud., GPT-2 Jud., RoBERTa Jud., and Sabiá Jud.) are significantly lower than those of their general-purpose counterparts. This finding underscores the value of domain-specific specialisation in improving clustering performance, as it leads to better-defined clusters. Notably, for the Sabiá Jud. model, the analysis clearly indicates that the optimal number of clusters is achieved with k=55.

"

Additionally, a new paragraph and a new table presenting the results obtained with 55 clusters have been added to the 'Results and Discussion' section."

Pages 14 lines 474-480

"

To ensure a fair comparison of the number of groups formed, the K obtained by the Sabiá Jud. technique was established as the standard, as it achieved the best result. Consequently, the grouping with K=55 was used for all techniques, as presented in Table 6. This approach consolidated the Sabiá Jud. model as the best for grouping legal documents using embeddings from transformer-based language models, ensuring consistency with the results presented earlier in Table 5.

Model	Mean	Std	Min	Q1	Q2	Q3	Max
Bbr	0.989	0.007	0.948	0.987	0.991	0.994	0.998
Bj	0.995	0.003	0.984	0.994	0.996	0.997	0.999
Gbr	0.988	0.008	0.963	0.987	0.990	0.993	0.998
Gj	0.995	0.004	0.979	0.994	0.996	0.997	0.998
Rbr	0.990	0.007	0.962	0.989	0.992	0.995	0.999
Rj	0.997	0.008	0.937	0.998	0.999	0.999	1.000
Sbr	0.850	0.135	0.532	0.744	0.866	0.997	1.000
Sj	0998	0.008	0.949	1.000	1.000	1.000	1.000

"

The authors should use more clustering techniques to do their experiments.

Answer: Thank you for your valuable comment. We have revised on page 15 of the "Results and Discussion" section to include an explanation of how names were assigned to the groups. This process provided semantic meaning to each cluster generated by the unsupervised learning technique, thereby improving both the interpretability and practical applicability of the clustering results.

Page 15 lines 527-544

"

To assist legal advisors in better identifying which TPUs are most related to each group, a simple and effective method for determining these terms was proposed. To provide semantic meaning to the groups, the most significant labels were assigned

to the 55 groups. This process involved extracting the three most relevant TPU terms from the documents within each group and using them as labels. For the technique that achieved the best performance, Sabiá Jud., embeddings of these TPU terms were calculated, and their cosine similarity to the group centroid was determined. The three terms with the highest cosine similarity were selected to name each group. Table 8 presents the group names defined by this technique for the groups with the best performance.

Group id	Amount of documents	Average similarity	Group name (TPUs)	Cosine similarity
7	49,638	1.000000	ctps	0.999804
			período de graça	0.999796
			astreintes	0.999795
8	2,689	0.999999	licença previdenciária	0.999999
			ação de cobrança	0.999999
			descontos previdenciários	0.999998
10	3,861	0.999999	licença previdenciária	0.999999
			suspeição	0.999998
			descontos previdenciários	0.999998
13	6,284	0.999999	agravo de instrumento	0.999999
			cesta básica	0.999999
			compensação em atividade insalubre	0.999999
16	3,785	0.999999	ente público	0.999999
			dirigente sindical	0.999999
			cálculo repercussão	0.999999
21	5,793	0.999999	seguro de vida	0.999998
			atraso na audiência	0.999997
			sucessão de empregadores	0.999997
23	5,947	0.999999	suspeição	0.999999
			licença previdenciária	0.999998
			deficiente físico	0.999998
24	4,962	0.999999	direito de greve	0.999999
			remuneração, verbas indenizatórias e benefícios	0.999999
			ação de cobrança	0.999999
26	2,709	0.999999	promoção	0.999999
			bancos	0.999999
			perícia	0.999999
28	4,825	0.999999	ação cautelar	0.999999
			compensação em atividade insalubre	0.999999
			tomador de serviços terceirização	0.999998
34	2,788	0.999999	salário família	0.999999
			salário in natura	0.999999

			extinção normal do contrato a termo	*0.999998*
38	*4,201*	*0.999999*	*deficiente físico*	*0.999999*
			engenheiro, arquiteto e engenheiro agrônomo	*0.999999*
			agravo de instrumento	*0.999999*
47	*5,250*	*0.999999*	*descontos previdenciários*	*0.999999*
			licença previdenciária	*0.999999*
			ação de cobrança	*0.999998*

Table 8. TPU terms most relevant to groups with average similarity greater than 0.999999, ordered by cosine similarity.

Based on Table 8, the high similarity between the most relevant terms and the centroid for each group stands out. This reinforces the quality of the groups generated by the technique, particularly in the context of the Labour Court, where this type of documents is typically associated with distinct terms. Additionally, it is noticed that in some groups, such as 8 and 47, the three terms are repeated, but in a different order. This means that, although their similarity levels are very close, they hold different semantic significance, which is important for decision-making processes taken by legal assessors during procedural evaluations. The full relation of groups and their most relevant TPUs based on this analysis can be found in Supporting information S1 Table.

"

They should analyze the clusters in detail.

Answer: Thank you for your valuable comment. This point was partially answered previously in item 1, however I repeat it here to facilitate review. A more detailed examination of the inertia plots was conducted, with a particular focus on the results obtained by the model using Sabiá Jud. The following text was added after the Inertia plots figure in the "Results and Discussion" section to provide further clarity:

Page 12 lines 432-441

"

The analysis of the inertia plots presented in Figure 8 reveals that, for all models except Sabiá Jud., the inertia generally decreased as the number of clusters increased, although with significant variance. An asymptotic downward trend can be observed through the dashed lines in these plots. Furthermore, the results emphasise that the inertia values obtained for all specialised models (BERT Jud., GPT-2 Jud., RoBERTa Jud., and Sabiá Jud.) are significantly lower than those of their general-purpose counterparts. This finding underscores the value of domain-specific specialisation in improving clustering performance, as it leads to better-defined clusters. Notably, for the Sabiá Jud. model, the analysis clearly indicates that the optimal number of clusters is achieved with k=55.

”

Additionally, to clarify the issue of the lack of labeled data and the motivation for using clustering rather than classification, a new paragraph was added on page 3 before Table 1 in the “Introduction” section. This addition explains the challenges associated with labeling data in the legal field and justifies the use of clustering techniques as an effective alternative for grouping similar documents, thereby optimising workflows and addressing one of the study's objectives.

Page 3 lines 59-69

“

> ***The Brazilian labour process is inherently complex, posing challenges for legal professionals in organising structured information related to various judicial matters within the electronic judicial system. A significant hurdle lies in the absence of labelled data, as labelling in the legal sector demands extensive hours of work from experts, whose time is both very limited and costly. Consequently, the lack of prior expert classification demands employing pattern detection through grouping techniques. This approach aligns with one of the primary motivations of this work: to support legal advisors by facilitating the grouping of similar documents, thereby optimising their workflow. Moreover, the groups formed using the methodology proposed in this research could, in the future, streamline the efforts of experts in labelling data, ultimately enabling the application of supervised learning techniques.***

“

Comparing with another research should be done on their experiments.

Answer: Thank you for your valuable comment. In this study, we developed an innovative methodology that, to the best of our knowledge, has not yet been published in any peer-reviewed journal. This work represents a pioneering effort in this area. Our research focuses on comparing transformer-based language models trained in Portuguese and specialised for a specific problem domain, whose vocabulary and context go beyond the general-purpose corpora dataset used to train these models, to enhance natural language pattern detection through an unsupervised learning technique. As no prior studies on this particular topic were identified, we lacked a direct basis for comparison or reference to guide our research. Furthermore, we found no previous work that utilised a dataset of this scale for such a specialised domain, or that conducted a comparative analysis of multiple transformer-based language models for pattern detection within this context. We hope this study will serve as a cornerstone for future research, establishing a benchmark for subsequent studies and fostering further advancements in this field.

In addition to the adjustments made based on your excellent comments and the reviewers' comments, we performed a complete review of the manuscript and decided to remove the

2D projection of the cluster from the Word2vec skip-gram to avoid copyright issues. This adjustment was made on page 15 lines 516-526.

We would like to thank you and the reviewers of this study who have provided valuable suggestions and very pertinent comments, thus helping to improve the quality of our paper.

Sincerely,

The authors.

Firstly, we would like to thank the comments and criticisms about our paper. Right now, we are ready to answer step by step all the major issues and minor corrections according to your requests. We hope these changes and explanations will satisfy all the raised points.

Reviewer #1:

This study offers an interesting and valuable contribution to the field of NLP. Overall, the writing quality is strong and clear. However, my primary concern is that the practical application of this work within the legal sector is not clearly articulated.

While the paper provides a thorough quantitative analysis of model performance, it lacks sufficient qualitative interpretation or insights into what these results truly signify in the context of legal document analysis. Given the nature of legal work, which is inherently qualitative, it would be beneficial to include a section that explores the practical relevance of the findings from a qualitative perspective.

Answer: Thank you for your valuable comment. The last paragraph of the "Results and Discussion" section has been revised, and a new paragraph has been added to present a qualitative analysis of the benefits that applying the methodology to a real-world case could provide.

Page 16-17 lines 545-564

> *"*
>
> ***Based on the methodology developed and evaluated in this work, a tool named GEMINI was created for the Brazilian Labour Court. This tool aids in searching for jurisprudence, distributing work among experts, and identifying opportunities to standardise legal interpretations within the courts, thereby establishing Cases of Uniformity of Jurisprudence. The GEMINI tool has been implemented across all twenty-four Brazilian Labour Courts, expediting the resolution of judicial cases in Brazilian Labour Court Justice [43].***
>
> ***The implementation of the tool in various Regional Labour Courts in Brazil has resulted in a significant reduction in the number of outstanding judicial cases. This supports the findings of this research, which indicate that grouping legal cases reduces the analysis time for assessors, thereby optimising workflow and ensuring that decisions uphold the principle of legal certainty [44-47]. Moreover, the methodology implemented through the GEMINI tool enabled faster analysis of legal proceedings by optimising the process of grouping documents, even in the absence of labels. This facilitated the work of legal advisors in a scenario of increasing demand within the Brazilian legal system, ensuring both the principle of legal certainty and the rapid processing of judicial proceedings. Furthermore, this approach contributes to advancing several Sustainable Development Goals (SDGs) defined by the United Nations, including Goal 8: "Decent Work and Economic***

Growth" and Goal 16: "Peace, Justice and Strong Institutions" specifically targets 8.3, 8.8, 16.3, and 16.6.

"

We would like to thank the reviewers of this study who have provided valuable suggestions and very pertinent comments, thus helping to improve the quality of our paper.

Sincerely,

The authors.

Firstly, we would like to thank the comments and criticisms about our paper. Right now, we are ready to answer step by step all the major issues and minor corrections according to your requests. We hope these changes and explanations will satisfy all the raised points.

Reviewer #2:

1 Good and useful idea.

Answer: Thank you so much.

2 Good introduction.

Answer: Thank you so much.

3 Good presentation of literature review.

Answer: Thank you so much.

4 It is preferable to use the passive voice when writing the paper.

Answer: Thank you for your valuable comment. We adjusted the text to the passive voice.

5 Figures lack high-resolution (not clear).

Answer: Thank you for your valuable comment. We ensured that all figures are generated at a resolution of 600 dpi.

6 Do you think that the dataset was sufficient to produce these results? Does reducing or increasing the number of words in the vector affect the results?

Answer: Thank you for your valuable comment.

Regarding the question concerning the size of the dataset, it represents approximately three years' worth of documents related to ordinary appeals filed in a medium-sized Brazilian Regional Labour Court. To clarify this point, the second-to-last paragraph of the "Introduction" section has been revised.

Page 4 lines 109-119

> *"*
>
> ***To delimit the scope of this research and enable a coherent comparison, the same dataset used in Oliveira and Sperandio Nascimento (2021) [14] was applied. The dataset includes approximately 210,000 Ordinary Appeals Filed (acronym in Portuguese for "Recurso Ordinário Interposto" - ROI) in legal proceedings[1], representing about three years worth of documents related to Ordinary Appeals Filed in a medium-sized Brazilian Regional Labour Court. The Ordinary Appeal was chosen as a reference because it is typically the document that escalates a case to a higher court (2nd degree), resulting in the Ordinary Appeal (acronym in Portuguese for "Recurso Ordinário" - RO). This appeal serves as a free plea, an appropriate***

[1] https://www.doi.org/10.5281/zenodo.7686233

appeal to final and terminating judgments rendered at first instance, seeking a review by a hierarchically superior judicial body [15]. Therefore, Figure 1 is included to illustrate the macro flow of the labour process.

"

Regarding the second question, which concerns whether the number of words in the vector affects the results, the following explanation was added before the last paragraph of the "Methodology" section to provide further clarity:

Page 11 lines 400-408

"

It is also worth noting that court documents typically contain a large number of words. NLP models with larger context windows, capable of handling more tokens at once, can easily accommodate entire documents in a single input sequence. This allows these models to fully capture the document's context without requiring text segmentation, unlike models with smaller context windows, as demonstrated in this research. When implementing this methodology, it is important to consider the size of the model's context window and determine whether text segmentation is necessary. In cases where the context window is smaller than the number of words in the document, the text segmentation technique proposed in this research is particularly appropriate.

"

7 It is preferable not to use references in the methodology or to reduce them. I think the methodology must be rewrite to be more understandable and readable.

Answer: Thank you for your valuable comment. The methodology was reviewed and we removed the references as far as we could to minimise the number of references in the methodology. However some references still remain there because they are necessary to explain where that information was coming from.

8 The references must be written in suitable style.

Answer: Thank you for your valuable comment. We thoroughly cross-checked the whole document to ensure compliance with the PLOS ONE LaTeX template in the current version of the manuscript.

We would like to thank the reviewers of this study who have provided valuable suggestions and very pertinent comments, thus helping to improve the quality of our paper.

Sincerely,

The authors.

Firstly, we would like to thank the comments and criticisms about our paper. Right now, we are ready to answer step by step all the major issues and minor corrections according to your requests. We hope these changes and explanations will satisfy all the raised points.

Reviewer #3:

The work "Analyzing similarities between legal court documents using natural language processing approaches based on Transformers" clusters 210,000 legal documents from the Brazilian justice system. The authors use k-means clustering on the document embeddings which they obtained by encoding the document using transformer-based models: BERT, RoBERTa, GPT-2, and Llama. The authors find that the average within-cluster document similarity increases when they use models fine-tuned to the legal domain.

My biggest concern with this work lies in the novelty of their contribution. NLP research has already proven that fine-tuning pre-trained models on the domain of interest will improve performance on downstream tasks. Therefore, we expect the "Jud"-version or specialized models to produce better semantic representations, and hence create more coherent clusters. That is not a novel finding.

Additionally, we also expect the contextualized embeddings to perform better than word2vec models because they are context-dependent. Therefore, the greater inter-cluster similarity scores in tables 3 and 4 over those in table 2 is not surprising.

Therefore, the only contribution of this paper is the application of transformer-based encoders to legal documents in the Brazilian Portuguese language. As the authors have pointed out, grouping the legal documents can assist the legal advisors in their work, as shown in Table 1. In that sense, this is more a systems paper than one doing novel methodological research. This is perfectly fine, and we do need more such works that apply conventional approaches to specialized domains and resource-constrained languages: legal Brazilian-Portuguese documents.

I suggest the authors clearly specify the contribution of this work. I recommend spending more time analyzing the clusters to find more interesting insights. For example, the authors say that Sabia produced better-defined clusters. It would be very interesting to see some examples of dissimilar documents that were clustered together by other models, but which Sabia correctly placed in different clusters, and vice-versa. Also, if the authors have some ground truth data, for example, information about legal proceedings that belong to the same group (maybe because they were deemed similar by legal advisors), they can perform an extrinsic evaluation of cluster purity.

Answer: Thank you for your valuable comment.

Regarding the question concerning the contribution of this work, the following explanation has been added to the "Introduction" section for further clarity:

Page 4 lines 120-131

"

Therefore, the main innovations of this work are:

- ***This research presents a novel analysis of the applicability of transformer-based models for clustering similar documents in the legal field.***
- ***Unsupervised learning was applied to identify patterns and determine the most effective and representative groups by clustering the embeddings generated by the transformer-based models for each legal document.***
- ***A comprehensive methodology was developed to ensure the reproducibility of results, enabling its application to not only the legal sector but also other specialised domain applications, for both the tested models and future transformer-based models.***
- ***The dataset used in this research has been made publicly available to support reproducibility and applicability to future studies in this field and beyond.***

"

Regarding the question concerning the analysis of the clusters to derive more meaningful insights, a more detailed examination of the inertia plots was conducted, with a particular focus on the results obtained by the model using Sabiá Jud. The following text was added after the Inertia plots figure in the "Results and Discussion" section to provide further clarity:

Page 12 lines 432-441

"

The analysis of the inertia plots presented in Figure 8 reveals that, for all models except Sabiá Jud., the inertia generally decreased as the number of clusters increased, although with significant variance. An asymptotic downward trend can be observed through the dashed lines in these plots. Furthermore, the results emphasise that the inertia values obtained for all specialised models (BERT Jud., GPT-2 Jud., RoBERTa Jud., and Sabiá Jud.) are significantly lower than those of their general-purpose counterparts. This finding underscores the value of domain-specific specialisation in improving clustering performance, as it leads to better-defined clusters. Notably, for the Sabiá Jud. model, the analysis clearly indicates that the optimal number of clusters is achieved with k=55.

"

Regarding the issue of selecting the best model, this choice was based on quantitative analysis using metrics derived from cosine distance, which are presented in the article and copied below for ease of reference.

Model	Grp	Mean	Std	Min	Q1	Q2	Q3	Max
Bbr	34	0.975	0.018	0.897	0.971	0.98	0.985	0.992
Bj	40	0.989	0.007	0.967	0.987	0.991	0.994	0.997
Gbr	38	0.974	0.018	0.933	0.966	0.98	0.986	0.995

Gj	32	0.989	**0.007**	**0.967**	0.988	0.991	0.993	0.998
Rbr	56	0.981	0.011	0.935	0.977	0.984	0.989	0.996
Rj	52	0.993	0.017	0.874	0.995	0.997	0.998	0.999
Sbr	113	0.727	0.253	0.000	0.529	0.768	0.995	1.000
Sj	55	**0.996**	0.016	0.899	**0.999**	**1.000**	**1.000**	**1.000**

Regarding the question concerning the qualitative analyses, we do not have labeled data indicating the correct class for each document, our evidence for the qualitative success of this methodology comes from the practical application of the GEMINI tool. This is demonstrated through official news articles, which are included in the manuscript for quick reference. These articles are:

- https://www.csjt.jus.br/web/csjt/justica-4-0/gemini
- https://www.trt5.jus.br/noticias/gemini-gabinetes-trt5-participam-projeto-piloto-que-utiliza-inteligencia-artificial
- https://www.trt5.jus.br/noticias/pje-trara-nova-versao-com-modulo-projeto-gemini-que-tem-participacao-trt5
- https://trt13.jus.br/informe-se/noticias/2021/07/trt-da-paraiba-integra-equipe-nacional-do-modulo-gemini

Although these news items are in Portuguese, they can be reliably translated into English using browser tools. The lack of labeled data presents a significant challenge, as labeling this data in the legal sector would require many hours of work from experts who are typically unavailable and whose time is costly. One of the main motivations for this work is to support legal advisors by facilitating the grouping of similar documents, thus optimising their workflow. So to clarify this issue of lack of labeled data, it was added one paragraph on page 3 before the Table 1 of the "Introduction" section.

Page 3 lines 59-69

> "
>
> ***The Brazilian labour process is inherently complex, posing challenges for legal professionals in organising structured information related to various judicial matters within the electronic judicial system. A significant hurdle lies in the absence of labelled data, as labelling in the legal sector demands extensive hours of work from experts, whose time is both very limited and costly. Consequently, the lack of prior expert classification demands employing pattern detection through grouping techniques. This approach aligns with one of the primary motivations of this work: to support legal advisors by facilitating the grouping of similar documents, thereby optimising their workflow. Moreover, the groups formed using the methodology proposed in this research could, in the future, streamline the efforts of experts in labelling data, ultimately enabling the application of supervised learning techniques.***
>
> "

I would also suggest that the authors should compare the clustering performance of the models on the same number of clusters (k). For any clustering approach, the average

inter-cluster similarity improves as the number of clusters increase. Therefore, it is unfair to compare the similarity scores between models if they cluster the documents into different number of clusters.

Answer: Thank you for your valuable comment. A new paragraph and a new table presenting the results obtained with 55 clusters have been added to the 'Results and Discussion' section."

Pages 14 lines 475-480

"

To ensure a fair comparison of the number of groups formed, the K obtained by the Sabiá Jud. technique was established as the standard, as it achieved the best result. Consequently, the grouping with K=55 was used for all techniques, as presented in Table 6. This approach consolidated the Sabiá Jud. model as the best for grouping legal documents using embeddings from transformer-based language models, ensuring consistency with the results presented earlier in Table 5.

Model	*Mean*	*Std*	*Min*	*Q1*	*Q2*	*Q3*	*Max*
Bbr	*0.989*	*0.007*	*0.948*	*0.987*	*0.991*	*0.994*	*0.998*
Bj	*0.995*	*0.003*	*0.984*	*0.994*	*0.996*	*0.997*	*0.999*
Gbr	*0.988*	*0.008*	*0.963*	*0.987*	*0.990*	*0.993*	*0.998*
Gj	*0.995*	*0.004*	*0.979*	*0.994*	*0.996*	*0.997*	*0.998*
Rbr	*0.990*	*0.007*	*0.962*	*0.989*	*0.992*	*0.995*	*0.999*
Rj	*0.997*	*0.008*	*0.937*	*0.998*	*0.999*	*0.999*	*1.000*
Sbr	*0.850*	*0.135*	*0.532*	*0.744*	*0.866*	*0.997*	*1.000*
Sj	*0998*	*0.008*	*0.949*	*1.000*	*1.000*	*1.000*	*1.000*

"

I also suggest that the authors provide more details of the fine-tuning process. They should tell us which objective functions they used. We would expect masked token training to be one of the objective functions. Did they also use next-sentence prediction? Fine-tuning encoder architectures such as BERT and RoBERTa is different from encoder-decoder architectures such as GPT-2 and Llama.

Answer: Thank you for your valuable comment. We reviewed the entire text in the "Fine-tuning of Transformer Models" section and incorporated a new paragraph and table to enhance clarity and improve the reader's understanding of the content.

Page 8-9 lines 291-314

"

In the learning process, depending on the Transformers model used, a token masking strategy is applied to each sentence. Masked Language Models (MLM) are utilised for BERT and RoBERTa models, while Causal Language Models (CLM) are employed for GPT-2 and LlaMA models. The CLM is trained unidirectionally to predict the next

word based on the preceding words [41], whereas the MLM employs a bidirectional approach to predict the masked words in the sentence.

Hence, for the training process of BERT models, simulations inspired by the article "Transformers: State-of-the-Art Natural Language Processing" [40] were conducted using masking rates of 15% and 25%. The results indicated that the 15% masking rate yielded the best performance in specialising the BERT model for Portuguese with the judicial corpus. Consequently, this masking rate was also applied to the RoBERTa model.

Finally, it is worth highlighting that it is essential to fine-tune the vocabulary using a specialised corpus. This process ensures that the model can incorporate new context more effectively, thereby enhancing its understanding of key terms. Additionally, fine-tuning helps the model grasp the specific language and jargon related to the content it will handle in NLP tasks.

Thus, as shown in Table 2, the fine-tuning of each model for the legal domain adhered to the same hyperparameters, tokenization techniques, and masking methods used during the training of their base models.

Model	Hiperparameters	Tokenization type	Masking method
BERT Jud.	24-layer, 1024-hidden, 16-heads, 340M parameters	word-piece	MLM
GPT-2 Jud.	12-layer, 768-hidden, 12-heads, 117M parameters	byte-level BPE	CLM
RoBERTa Jud.	12-layer, 768-hidden, 12-heads, 125M parameters	byte-level BPE	MLM
Sabiá Jud.	32-layer, 4096-hidden, 32-heads, 6.7B parameters	sentence-piece BPE	CLM

Thus, after fine-tuning the models to create specialised NLP models for the legal field, as well as adapting pre-existing models in Portuguese, it was necessary to calculate the vector representations of the documents. This process is detailed in the following section. Subsequently, document embeddings for each of the eight models were used in an unsupervised training technique to generate the clusters.

"

We would like to thank the reviewers of this study who have provided valuable suggestions and very pertinent comments, thus helping to improve the quality of our paper.

Sincerely,

The authors.

Firstly, we would like to thank the comments and criticisms about our paper. Right now, we are ready to answer step by step all the major issues and minor corrections according to your requests. We hope these changes and explanations will satisfy all the raised points.

Reviewer #4:

In this paper, the authors developed a legal document clustering pipeline, in order to support efficient case distributing and reviewing. Transformer-based language models were trained to generate domain-specific contextual word embedding and compute document embedding, which were further used for the k-means clustering of legal documents. The authors implemented the proposed pipeline on a large, real-world law case data set, and compared their performance with traditional NLP and embedding methods. The background was stated in detail. As the traditional manual review for law cases requires extensive time and effort, AI-based models have good potential in real-world application. Both encoder-only and decoder-only transformer architectures were considered in this study, which provides a comprehensive comparison. Despite the strength mentioned above, I have the following questions / concerns.

1) Document grouping mechanism. In the Introduction section, the authors mentioned that grouping cases with similar requests can improve the efficiency. Why was clustering chosen rather than classification? By labeling typical types of such requests as different case classes, deep document classifiers can be fine-tuned in a supervised manner to gain more accurate and interpretable predictions of classes (groups). Different levels of classes can be designed to train multiple classifiers as well. By having the class information, certain types of cases can be assigned to those team members with specific domain expertise. If clustering is used, extra effort will be needed to interpret each cluster of cases. The authors need to better explain the motivation and real-world advantages of using clustering rather than classification in this application domain.

Answer: Thank you for your valuable comment. To clarify the issue of the lack of labeled data and the motivation for using clustering rather than classification, a new paragraph was added on page 3 before Table 1 in the “Introduction” section. This addition explains the challenges associated with labeling data in the legal field and justifies the use of clustering techniques as an effective alternative for grouping similar documents, thereby optimising workflows and addressing one of the study's objectives.

Page 3 lines 59-69

> *“*
>
> ***The Brazilian labour process is inherently complex, posing challenges for legal professionals in organising structured information related to various judicial matters within the electronic judicial system. A significant hurdle lies in the absence of labelled data, as labelling in the legal sector demands extensive hours of work from experts, whose time is both very limited and costly. Consequently, the lack of prior expert classification demands employing pattern detection through grouping***

techniques. This approach aligns with one of the primary motivations of this work: to support legal advisors by facilitating the grouping of similar documents, thereby optimising their workflow. Moreover, the groups formed using the methodology proposed in this research could, in the future, streamline the efforts of experts in labelling data, ultimately enabling the application of supervised learning techniques.

"

2) Cluster interpretation results and discussions. One key step in a clustering pipeline is to interpret the meaning of clusters, which is missing in this study. The authors chose from 30 to 61 as the number of clusters, does each cluster have practical meaning (e.g., a specific type of request, as mentioned in the Introduction)? Related to 1), clustering results are not naturally interpretable as classification, and it is very important to discuss the semantic meaning of each cluster, in addition to reporting the similarity.

Answer: Thank you for your valuable comment. We have revised on page 15 of the "Results and Discussion" section to include an explanation of how names were assigned to the groups. This process provided semantic meaning to each cluster generated by the unsupervised learning technique, thereby improving both the interpretability and practical applicability of the clustering results.

Page 15 lines 527-544

"

To assist legal advisors in better identifying which TPUs are most related to each group, a simple and effective method for determining these terms was proposed. To provide semantic meaning to the groups, the most significant labels were assigned to the 55 groups. This process involved extracting the three most relevant TPU terms from the documents within each group and using them as labels. For the technique that achieved the best performance, Sabiá Jud., embeddings of these TPU terms were calculated, and their cosine similarity to the group centroid was determined. The three terms with the highest cosine similarity were selected to name each group. Table 8 presents the group names defined by this technique for the groups with the best performance.

Group id	*Amount of documents*	*Average similarity*	*Group name (TPUs)*	*Cosine similarity*
7	*49,638*	*1.000000*	*ctps*	*0.999804*
			período de graça	*0.999796*
			astreintes	*0.999795*
8	*2,689*	*0.999999*	*licença previdenciária*	*0.999999*
			ação de cobrança	*0.999999*
			descontos previdenciários	*0.999998*
10	*3,861*	*0.999999*	*licença previdenciária*	*0.999999*
			suspeição	*0.999998*
			descontos previdenciários	*0.999998*

13	6,284	0.999999	agravo de instrumento	0.999999
			cesta básica	0.999999
			compensação em atividade insalubre	0.999999
16	3,785	0.999999	ente público	0.999999
			dirigente sindical	0.999999
			cálculo repercussão	0.999999
21	5,793	0.999999	seguro de vida	0.999998
			atraso na audiência	0.999997
			sucessão de empregadores	0.999997
23	5,947	0.999999	suspeição	0.999999
			licença previdenciária	0.999998
			deficiente físico	0.999998
24	4,962	0.999999	direito de greve	0.999999
			remuneração, verbas indenizatórias e benefícios	0.999999
			ação de cobrança	0.999999
26	2,709	0.999999	promoção	0.999999
			bancos	0.999999
			perícia	0.999999
28	4,825	0.999999	ação cautelar	0.999999
			compensação em atividade insalubre	0.999999
			tomador de serviços terceirização	0.999998
34	2,788	0.999999	salário família	0.999999
			salário in natura	0.999999
			extinção normal do contrato a termo	0.999998
38	4,201	0.999999	deficiente físico	0.999999
			engenheiro, arquiteto e engenheiro agrônomo	0.999999
			agravo de instrumento	0.999999
47	5,250	0.999999	descontos previdenciários	0.999999
			licença previdenciária	0.999999
			ação de cobrança	0.999998

Table 8. TPU terms most relevant to groups with average similarity greater than 0.999999, ordered by cosine similarity.

Based on Table 8, the high similarity between the most relevant terms and the centroid for each group stands out. This reinforces the quality of the groups generated by the technique, particularly in the context of the Labour Court, where this type of documents is typically associated with distinct terms. Additionally, it is noticed that in some groups, such as 8 and 47, the three terms are repeated, but in a different order. This means that, although their similarity levels are very close, they hold different semantic significance, which is important for decision-making

processes taken by legal assessors during procedural evaluations. The full relation of groups and their most relevant TPUs based on this analysis can be found in Supporting information S1 Table.

"

3) Data set. The paragraph of Line 98 introduces the large data set used, and it is important to illustrate the statistics of the data in detail. For example, roughly how many types of legal requests or cases are there in this data set? Do these types align with the cluster number (30-61) the authors selected? Document length is important in this study as the authors carefully designed the segmenting methods. What is the average document token number in this data set? A histogram of documents with respect to token number can help readers understand the characteristics of data. Such key statistics above are missing in the manuscript.

Answer: Thank you for your valuable comment.

Regarding the question concerning the types of legal requests or cases are there in this data set. In this problem domain labeled data is unavailable, then we defined the scope of documents to be grouped as those of the type "Filed Ordinary Appeal". To better clarify this for readers, a new paragraph has been added on page 3 before Table 1 in the “Introduction” section.

Page 3 lines 59-69

"

The Brazilian labour process is inherently complex, posing challenges for legal professionals in organising structured information related to various judicial matters within the electronic judicial system. A significant hurdle lies in the absence of labelled data, as labelling in the legal sector demands extensive hours of work from experts, whose time is both very limited and costly. Consequently, the lack of prior expert classification demands employing pattern detection through grouping techniques. This approach aligns with one of the primary motivations of this work: to support legal advisors by facilitating the grouping of similar documents, thereby optimising their workflow. Moreover, the groups formed using the methodology proposed in this research could, in the future, streamline the efforts of experts in labelling data, ultimately enabling the application of supervised learning techniques.

"

Regarding the question concerning the cluster number (30-61). Since labeled data is unavailable, it was not possible to directly compare the number of groups in the dataset with the number of groups selected. Instead, the value of K was systematically determined by testing a range of K values and analysing inertia using the elbow method to identify the optimal K. This approach enabled the use of unsupervised learning to extract patterns from the data without prior knowledge of these patterns. Consequently, the research introduced an innovative methodology to explore and analyze the data, marking a novel application in

this domain. Then, we tested thirty-one values for K, ranging from 30 to 61 in unit intervals, and the optimal K was selected based on the elbow method and a more detailed examination of the inertia plots was conducted, with a particular focus on the results obtained by the model using Sabiá Jud. The following text was added after the Inertia plots figure in the "Results and Discussion" section to provide further clarity:

Page 12 lines 432-441

> *"*
>
> ***The analysis of the inertia plots presented in Figure 8 reveals that, for all models except Sabiá Jud., the inertia generally decreased as the number of clusters increased, although with significant variance. An asymptotic downward trend can be observed through the dashed lines in these plots. Furthermore, the results emphasise that the inertia values obtained for all specialised models (BERT Jud., GPT-2 Jud., RoBERTa Jud., and Sabiá Jud.) are significantly lower than those of their general-purpose counterparts. This finding underscores the value of domain-specific specialisation in improving clustering performance, as it leads to better-defined clusters. Notably, for the Sabiá Jud. model, the analysis clearly indicates that the optimal number of clusters is achieved with k=55.***
>
> *"*

Regarding the question concerning the document token number in this data set, we added a new figure in the "Methodology" section to illustrate the number of words per document.

Page 9-10 lines 336-346

> *"*
>
> ***As indicated in Section State-of-the-Art Review, unlike the present work, which utilises very large documents, current research typically limits the texts used in vector transformations to the maximum number of words supported by the respective architecture. Figure 6 shows a histogram and distribution plot, highlighting that most documents in the dataset contain up to approximately 3,500 words, with an average of 2,525 words. Additionally, documents exceeding 10,000 words are considered outliers in the distribution, totalling 13,661 documents. Despite this they were still considered in this research. It is important to note that the models considered in this research have input size limits ranging from 512 tokens (BERT) to 4096 tokens (Sabiá). Therefore, the approach used in this study was crucial to enable the models to process more tokens than their maximum input size at one time.***

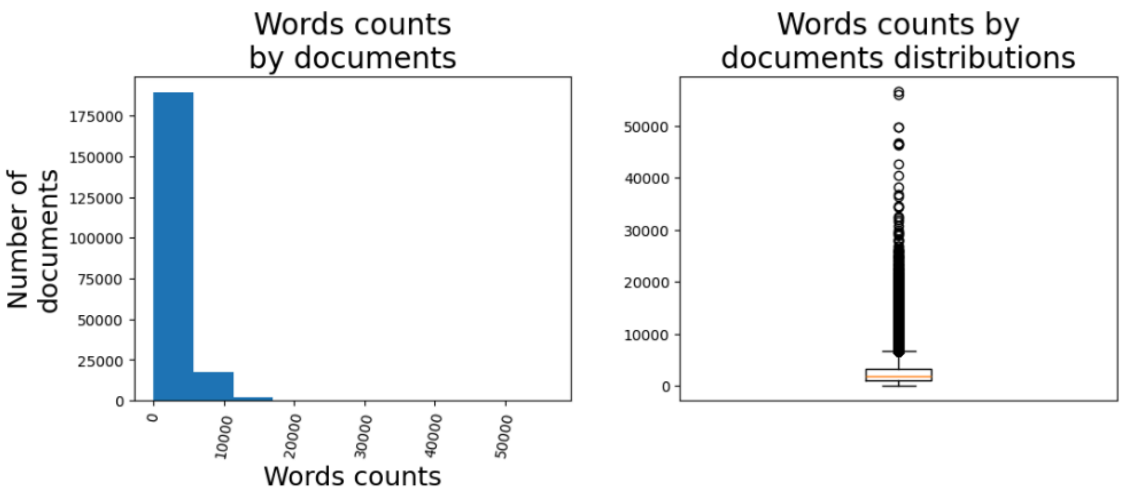

"

4) Language model. Llama 3.1, 3.2 and 3.3 series all have pre-trained LLMs with 128K context windows. Specifically, Llama 3.1 has 8B (https://huggingface.co/meta-llama/Llama-3.1-8B) version and 3.2 has 1B/3B versions which are of the same size level as the models authors used in this study. These new models are multilingual and natively support Portuguese. Related to 3), if the majority of those legal documents have less than 128K tokens, do we still need to segment the document as proposed in the paper, when we choose these latest models or future models with even larger context windows?

Answer: Thank you for your valuable comment.

Regarding the question concerning whether the context windows, in this study, we did not use the most modern models because they were unavailable at the time of the research, and their hardware requirements significantly exceeded the resources available for this work. However, it is worth noting that the proposed methodology can be easily extended to accommodate other language models in the future. Additionally, the following explanation was added before the last paragraph of the "Methodology" section to provide further clarity:

Page 11 lines 400-408

"

It is also worth noting that court documents typically contain a large number of words. NLP models with larger context windows, capable of handling more tokens at once, can easily accommodate entire documents in a single input sequence. This allows these models to fully capture the document's context without requiring text segmentation, unlike models with smaller context windows, as demonstrated in this research. When implementing this methodology, it is important to consider the size of the model's context window and determine whether text segmentation is necessary. In cases where the context window is smaller than the number of words in

the document, the text segmentation technique proposed in this research is particularly appropriate.

"

Regarding the question concerning the Llama 3.1, 3.2, and 3.3 series, we clarify that this research focuses on NLP models specifically trained with Portuguese-language data, developed in prior scientific studies, and made publicly available. Consequently, more recent models, such as Llama 3, trained on multilingual data, may not be ideal for this type of application due to their training data being predominantly English-language content (95%), with only a small proportion (5%) allocated to other languages, including Portuguese (https://ai.meta.com/blog/meta-llama-3/).

5) Clustering method. The authors selected k-means as the clustering method. While k-means is commonly used, other clustering methods may have extra advantages in this application. Hierarchical clustering can group the cases in different levels of clusters, which naturally fits the layered relationships and nested categorizations in the legal domain. Spatial clustering methods such as DBSCAN can naturally filter out and identify noise or unusual law cases, which can be helpful in large and varied legal data. HDBSCAN incorporates both advantages above. Related to 1), the authors need to add more clarification about why k-means is chosen and how to interpret the clusters, given that there are more clustering methods with extra benefits in this application scenario.

Answer: Thank you for your very valuable comment - we really appreciated it.

We utilised various transformer-based language models and a well-known clustering technique to demonstrate the behavior of each model in the unsupervised analysis process. Although we aimed to explore additional techniques, such as HDBSCAN, we encountered limitations due to the volume of data and the significant hardware requirements, which exceeded our available resources. For this research we use a powerful server: Intel(R) Xeon(R) Gold 6148 CPU @ 2.40GHz with 40 CPUs and approximately 376 GB of RAM.

Following your suggestion, we attempted to apply HDBSCAN. However, we encountered limitations due to the volume of data and the significant hardware requirements needed to run the technique. Despite our efforts, we were only able to process half of the data within the available computational environment. Moreover, the results indicated, as shown in the Figure below, that the technique classified a large majority of the data as anomalous, which contradicts the nature of the dataset, as it consists of regular legal proceedings within the labour courts.

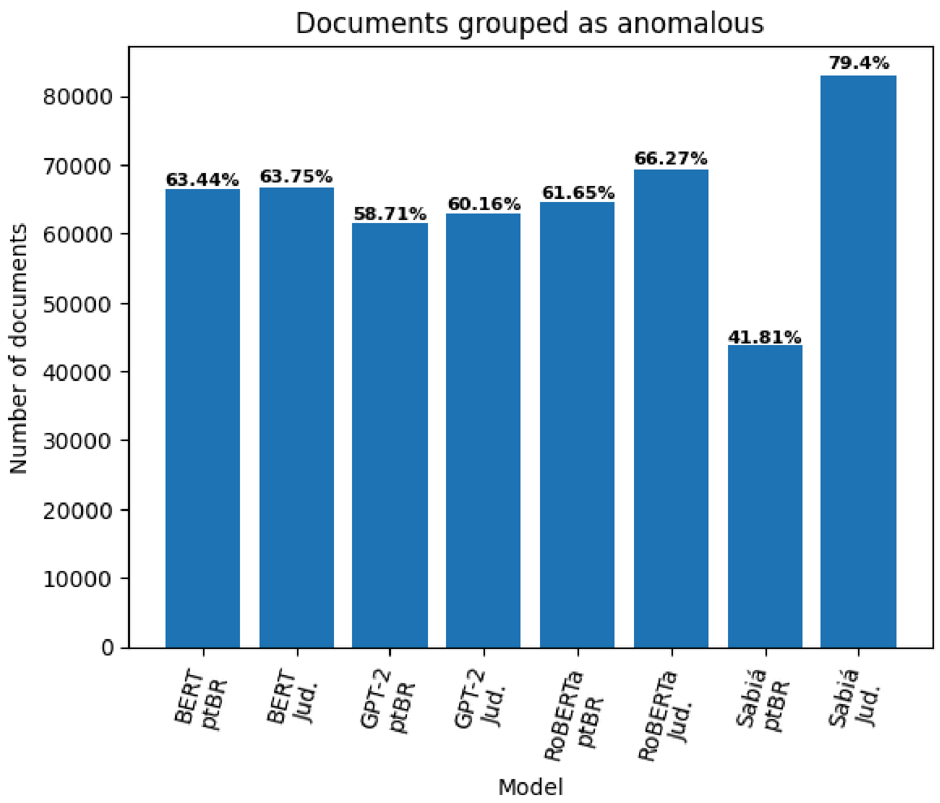

We chose not to include these results in the manuscript because we could not process the full dataset with the available hardware, which would not align with the objectives of this research. Nonetheless, the partial application of HDBSCAN provided valuable insights. It demonstrated that our proposed methodology allows flexibility for researchers to explore and apply alternative techniques, making it straightforward to substitute one approach for another.

Our primary focus is to show that a standard unsupervised learning technique can effectively yield meaningful results in this context. We hope that future work in the scientific community will build on this foundation, bringing new perspectives and exploring alternative methods. To facilitate such contributions, we are making the dataset publicly available.

We have revised the last paragraph of the "Conclusions and Future Works" section to bring these possibilities of future work.

Page 18 lines 601-613

> *"*
>
> ***Therefore, future work should focus on fine-tuning a large language model (LLM) in Portuguese, such as LLaMA, by specialising its vocabulary before training, or alternatively, training a generalist LLM model in Portuguese from scratch to compare their performance in real-case scenarios. Additionally, opportunities exist to validate the word embeddings generated in this study for other types of legal documents and***

domains, as well as to apply them in various tasks, such as generating decision drafts and classifying documents and legal cases.

Exploring faster techniques for transforming text into vector representations is also recommended to enhance efficiency. Furthermore, the proposed methodology provides flexibility, allowing researchers to explore and apply alternative techniques, making it straightforward to substitute one approach for another. This adaptability enables the scientific community to build on this foundation, offering new perspectives and exploring alternative methods.

"

6) In Figure 7 result, the Sabia 7B Jud. curve is a bit strange, as one specific cluster number is significantly better than all other numbers. Why is this number special? It would be valuable to interpret this specific case.

Answer: Thank you for your valuable comment. The inertia plot figure was adjusted and a paragraph was added after the figure with an analysis of the inertia plots.

Page 12 lines 432-441

"

The analysis of the inertia plots presented in Figure 8 reveals that, for all models except Sabiá Jud., the inertia generally decreased as the number of clusters increased, although with significant variance. An asymptotic downward trend can be observed through the dashed lines in these plots. Furthermore, the results emphasise that the inertia values obtained for all specialised models (BERT Jud., GPT-2 Jud., RoBERTa Jud., and Sabiá Jud.) are significantly lower than those of their general-purpose counterparts. This finding underscores the value of domain-specific specialisation in improving clustering performance, as it leads to better-defined clusters. Notably, for the Sabiá Jud. model, the analysis clearly indicates that the optimal number of clusters is achieved with k=55.

"

7) Writing. Things like the Python version (Line 192) should go to Experiment or Result section, as they are implementation details rather than Methodology. In Line 383, "Using" should be in lowercase.

Answer: Thank you for your valuable comment. Adjustments were made accordingly.

We would like to thank the reviewers of this study who have provided valuable suggestions and very pertinent comments, thus helping to improve the quality of our paper.

Sincerely,

The authors.
