# Supplementary material for: Analysing similarities between legal court documents using natural language processing approaches based on transformers
Source: PLoS One. 2025 Apr 8;20(4):e0320244. doi: 10.1371/journal.pone.0320244 (PMC11978053; doi:10.1371/journal.pone.0320244)
Supplement: Attachment — Submitted filename: review1.docx [file pone.0320244.s009.docx]

Manuscript Number: PONE-D-24-49879

**Analysing similarities between legal court documents using natural language processing approaches based on Transformers**

1- Good and useful idea.
2- Good introduction.
3- Good presentation of literature review.
4- It is preferable to use the passive voice when writing the paper.
5- Figures lack high-resolution (not clear).
6- Do you think that the dataset was sufficient to produce these results? Does reducing or increasing the number of words in the vector affect the results?
7- It is preferable not to use references in the methodology or to reduce them. I think the methodology must be rewrite to be more understandable and readable.
8- The references must be written in suitable style.
